# Supplementary material for: Modeling glioblastoma heterogeneity as a dynamic network of cell states
Source: Mol Syst Biol. 2021 Sep 16;17(9):e10105. doi: 10.15252/msb.202010105 (PMC8444284; doi:10.15252/msb.202010105)
Supplement: Supplementary file 6 — Source Data for Figure 5 [file MSB-17-e10105-s004.zip › Figure5A_sourcedata/GSEA_3071/hallmarks_stateII.GseaPreranked.1623742536692/HALLMARK_HEME_METABOLISM.html]

Details for gene set HALLMARK\_HEME\_METABOLISM[GSEA]

|  || Dataset | state23071 |
| Phenotype | NoPhenotypeAvailable |
| Upregulated in class | na\_neg |
| GeneSet | HALLMARK\_HEME\_METABOLISM |
| Enrichment Score (ES) | -0.3384962 |
| Normalized Enrichment Score (NES) | -1.6503638 |
| Nominal p-value | 0.012152778 |
| FDR q-value | 0.11079061 |
| FWER p-Value | 0.425 |
Table: GSEA Results Summary

  

Fig 1: Enrichment plot: HALLMARK\_HEME\_METABOLISM      
 Profile of the Running ES Score & Positions of GeneSet Members on the Rank Ordered List

  

| PROBE | GENE SYMBOL | GENE\_TITLE | RANK IN GENE LIST | RANK METRIC SCORE | RUNNING ES | CORE ENRICHMENT || 1 | CCND3 |  |  | 390 | 0.683 | -0.0707 | No |
| 2 | SLC2A1 |  |  | 787 | 0.503 | -0.1497 | No |
| 3 | BCAM |  |  | 816 | 0.493 | -0.1381 | No |
| 4 | ELL2 |  |  | 837 | 0.489 | -0.1246 | No |
| 5 | OSBP2 |  |  | 922 | 0.466 | -0.1279 | No |
| 6 | SLC7A11 |  |  | 998 | 0.445 | -0.1297 | No |
| 7 | GMPS |  |  | 1126 | 0.413 | -0.1455 | No |
| 8 | MAP2K3 |  |  | 1136 | 0.410 | -0.1323 | No |
| 9 | UCP2 |  |  | 1138 | 0.410 | -0.1171 | No |
| 10 | MFHAS1 |  |  | 1139 | 0.410 | -0.1017 | No |
| 11 | P4HA2 |  |  | 1398 | 0.358 | -0.1520 | No |
| 12 | HEBP1 |  |  | 1484 | 0.342 | -0.1601 | No |
| 13 | NR3C1 |  |  | 1510 | 0.339 | -0.1535 | No |
| 14 | CDC27 |  |  | 1562 | 0.330 | -0.1537 | No |
| 15 | BMP2K |  |  | 1824 | 0.296 | -0.2071 | No |
| 16 | BPGM |  |  | 1832 | 0.294 | -0.1978 | No |
| 17 | TSPAN5 |  |  | 1861 | 0.291 | -0.1938 | No |
| 18 | GCLM |  |  | 1889 | 0.287 | -0.1896 | No |
| 19 | NEK7 |  |  | 2048 | 0.267 | -0.2187 | No |
| 20 | BLVRA |  |  | 2152 | 0.257 | -0.2345 | No |
| 21 | NFE2L1 |  |  | 2302 | -0.259 | -0.2616 | No |
| 22 | CAT |  |  | 2344 | -0.264 | -0.2618 | No |
| 23 | GDE1 |  |  | 2616 | -0.303 | -0.3174 | No |
| 24 | DCUN1D1 |  |  | 2667 | -0.311 | -0.3180 | No |
| 25 | MKRN1 |  |  | 2669 | -0.312 | -0.3066 | No |
| 26 | LAMP2 |  |  | 2724 | -0.321 | -0.3078 | No |
| 27 | BSG |  |  | 2844 | -0.343 | -0.3243 | No |
| 28 | ADD1 |  |  | 2886 | -0.353 | -0.3212 | No |
| 29 | RBM38 |  |  | 2957 | -0.366 | -0.3247 | Yes |
| 30 | MXI1 |  |  | 3000 | -0.372 | -0.3211 | Yes |
| 31 | CIR1 |  |  | 3007 | -0.375 | -0.3085 | Yes |
| 32 | ATP6V0A1 |  |  | 3014 | -0.378 | -0.2957 | Yes |
| 33 | ARHGEF12 |  |  | 3022 | -0.378 | -0.2832 | Yes |
| 34 | ALDH6A1 |  |  | 3027 | -0.379 | -0.2699 | Yes |
| 35 | UROS |  |  | 3092 | -0.396 | -0.2708 | Yes |
| 36 | RNF19A |  |  | 3137 | -0.411 | -0.2662 | Yes |
| 37 | AGPAT4 |  |  | 3140 | -0.411 | -0.2512 | Yes |
| 38 | NARF |  |  | 3223 | -0.434 | -0.2552 | Yes |
| 39 | SDCBP |  |  | 3257 | -0.442 | -0.2467 | Yes |
| 40 | RBM5 |  |  | 3381 | -0.490 | -0.2586 | Yes |
| 41 | NUDT4 |  |  | 3421 | -0.510 | -0.2491 | Yes |
| 42 | PGLS |  |  | 3432 | -0.513 | -0.2322 | Yes |
| 43 | KLF3 |  |  | 3481 | -0.533 | -0.2240 | Yes |
| 44 | ADD2 |  |  | 3492 | -0.538 | -0.2063 | Yes |
| 45 | LMO2 |  |  | 3536 | -0.563 | -0.1957 | Yes |
| 46 | FOXO3 |  |  | 3594 | -0.601 | -0.1872 | Yes |
| 47 | YPEL5 |  |  | 3603 | -0.605 | -0.1663 | Yes |
| 48 | TFDP2 |  |  | 3637 | -0.634 | -0.1507 | Yes |
| 49 | HAGH |  |  | 3641 | -0.636 | -0.1274 | Yes |
| 50 | GYPC |  |  | 3722 | -0.701 | -0.1208 | Yes |
| 51 | CLCN3 |  |  | 3734 | -0.711 | -0.0968 | Yes |
| 52 | TNRC6B |  |  | 3781 | -0.771 | -0.0791 | Yes |
| 53 | DAAM1 |  |  | 3856 | -0.849 | -0.0654 | Yes |
| 54 | IGSF3 |  |  | 3886 | -0.912 | -0.0382 | Yes |
| 55 | BTG2 |  |  | 3917 | -0.999 | -0.0080 | Yes |
| 56 | PRDX2 |  |  | 4021 | -1.396 | 0.0190 | Yes |
Table: GSEA details [plain text format]

  

Fig 2: HALLMARK\_HEME\_METABOLISM: Random ES distribution      
 Gene set null distribution of ES for **HALLMARK\_HEME\_METABOLISM**
